# Geographical variation in compulsory hospitalisation – ethical challenges

**DOI:** 10.1186/s12913-022-08798-2

**Published:** 2022-12-10

**Authors:** Tore Hofstad, Tonje Lossius Husum, Jorun Rugkåsa, Bjørn Morten Hofmann

**Affiliations:** 1 Centre for Medical Ethics, University of Oslo, Oslo, Norway; 2 Faculty of Health Sciences, Oslo Metropolitan University, Oslo, Norway; 3 Health Services Research Unit, Akershus University Hospital, Lørenskog, Norway; 4 Centre for Care Research, University of South-Eastern Norway, Porsgrunn, Norway; 5 Department of Health Sciences, Norwegian University of Science and Technology, Gjøvik, Norway

**Keywords:** Service delivery variation, Involuntary hospitalisation, Coercion, Small area analysis, Autonomy, Beneficence, Non-maleficence, Justice, Right care, Ethical analysis

## Abstract

**Background:**

Compulsory hospitalisation in mental health care restricts patients’ liberty and is experienced as harmful by many. Such hospitalisations continue to be used due to their assumed benefit, despite limited scientific evidence. Observed geographical variation in compulsory hospitalisation raises concern that rates are higher and lower than necessary in some areas.

**Methods/discussion:**

We present a specific normative ethical analysis of how geographical variation in compulsory hospitalisation challenges four core principles of health care ethics. We then consider the theoretical possibility of a “right”, or appropriate, level of compulsory hospitalisation, as a general norm for assessing the moral divergence, i.e., too little, or too much. Finally, we discuss implications of our analysis and how they can inform the future direction of mental health services.

## Introduction

Geographical variation in health care delivery raises concerns about equity and efficiency of services [1], and it is considered one of the most important topics in health services research [2]. Geographical variations can indicate overuse and underuse of health services [3]. Overuse describes unnecessary or ineffective health care where the benefits do not outweigh potential harm. Underuse represents the failure to provide necessary, effective care. Geographical variation in service delivery has been observed in a wide range of areas in medicine and health care [1]. Examples from mental health care [4] include the use of electroconvulsive therapy [5]; dimensioning of services [6]; prevalence or diagnosing of mental disorders [7–9]; medication use [10, 11]; community treatment orders [12, 13]; and voluntary psychiatric admissions [14, 15]; discharges [16]; and readmissions [17].

Health care delivery is based on informed consent. Compulsory hospitalisation in mental health care constitutes an exception as it is enforced without the patients’ consent. Moreover, it restricts their liberty [18] and is by many experienced as an intrusion or abuse [19]. For legal and ethical reasons, compulsory hospitalisation should be a last resort after voluntary options have been exhausted. There is growing evidence that voluntary approaches, such as Assertive Community Treatment, can contribute to strengthening patient autonomy while reducing the need for compulsory hospitalisation [18, 20]. A recent compendium from the Council of Europe contains examples from fifteen countries of good practices to promote voluntary initiatives that have reduced coercion and the use of compulsory hospitalisation [21].

Criteria for compulsory hospitalisation differ between legislations, but are typically restricted to persons with severe mental illness (SMI) who represent a danger to themselves or others, or are in need of treatment to prevent serious deterioration of their mental or physical health [22]. Compulsory hospitalisations are usually only permitted if voluntariness has failed, or is clearly futile, and the admission is perceived to be in the patient’s best interest. The exception to this is if there is serious and imminent risk to the life or health of others. Following the United Nations’ Convention on the Rights of Persons with Disabilities (CRPD) [23, 24], the ethics of compulsory hospitalisation has increasingly been discussed in terms of patient rights and human rights. The convention highlights that persons with mental and/or physical disabilities should have equal rights to freely accept or reject health services. In response to the CRPD, several countries have amended their mental health legislations to incorporate supported decision-making to ensure that patients are allowed to express their wills and preferences [24]. Despite political initiatives and sustained attempts at reducing compulsory hospitalisation, its use remains widespread and is increasing in many countries [25].

Studies of geographical variation in health care often investigate areas that are legally, epidemiologically, or demographically comparable. Variation that can be accounted for by factors known to impact the issue under study - for instance age, gender, area morbidity or disease prevalence - is usually not considered problematic. Analyses are therefore conventionally performed on population-based rates that are risk-adjusted. The term unwarranted variation is used to describe variation that is unexplained by such warranting factors. The existence of considerable geographic variation in compulsory hospitalisation, beyond that which can be expected based on known risk factors [26], suggests that the supply and organisation of services may contribute to this variation, or that practise is based more on experience than evidence [27]. Unwarranted variation raises concern that coercion is used more than strictly necessary in some areas [28]. However, concern has also been voiced that too low rates of coercion can fail to meet patient needs and result in serious mental or physical harm [29–31].

Geographical variation in compulsory hospitalisation has been documented in several countries, including Denmark [32], England [33, 34], Finland [35], France [36], Germany [37], Ireland [38], Italy [39], Korea [40], the Netherlands [41], New Zealand [42], Sweden [43], Switzerland [44], and the United States [45]. In Norway between 2014 and 2018, the average rate of compulsory hospitalisation in the highest ranked catchment area was 5.6 times higher than the rate in the lowest ranked area, and the number of days of compulsory hospitalisation was eight times higher [46]. Compared to other health services [1], the observed geographical variation in compulsory hospitalisation was considered high to very high [46].

The ethics of compulsory hospitalisation have been debated for a long time [47–56]. Topics include the tension between principles of autonomy and beneficence; between a focus on self-determination and civil liberties as argued from a deontological position, versus patients’ right to treatment as perceived from a paternalistic viewpoint, primarily motivated by consequentialist reasoning [52]. Furthermore, controversy arises due to the evidence of harm to patients resulting from compulsory hospitalisation [57]. There is also disagreement on the use of compulsory hospitalisation for public safety when there is risk of harm to others [47]. To date, no ethical analysis of geographical variation in compulsory hospitalisation has been published. Therefore, this is the focus of our article.

### Methods

First, we report a normative ethical analysis structured according to four core principles of health care ethics [58], to identify how they may be challenged by geographical variation in compulsory hospitalisation. We define an ethical challenge [59] in this context as a situation where an ethical principle is infringed, where two or more principles appear to be in conflict, or where there is uncertainty about what constitutes “right care” (see below).

Principlism has been prominent within health care ethics since the Belmont report in 1978 and the first edition of Beauchamp and Childress’ book Principles of Biomedical Ethics in 1979 [58]. According to Beauchamp and Childress, four principles can be derived from a common morality and have wide applicability. They are summarised as follows: “respect for autonomy (the obligation to respect the decision making capacities of autonomous persons); non-maleficence (the obligation to avoid causing harm); beneficence (obligations to provide benefits and to balance benefits against risks), and justice (obligations of fairness in the distribution of benefits and risks)” ([60] p269). The principles are not hierarchically ordered and should be considered in combination.

Second, to facilitate reflections on overuse and underuse, we consider the theoretical possibility of a “right”, or appropriate, level of compulsory hospitalisation. Finally, we discuss implications of our analysis and how they can inform mental health services.

## Ethical analysis using the ‘four principles’ model

### Respect for autonomy

The principle of respecting autonomy implies that people have the right to make independent decisions about their lives, including the right to refuse health care. Compulsory hospitalisation appears to fundamentally conflict with this principle [61]. Thus, geographical variations in compulsory hospitalisation are ethically challenging as they indicate variation in respect for autonomy.

There are, however, three requirements for autonomy according to Beauchamp and Childress’ model: capacity for autonomous decisions, understanding, and voluntariness [58], each of which could be impeded by SMI. If a person lacks decision-making capacity, the person is not considered autonomous within this framework. Likewise, SMI can impair a person’s capacity for understanding [62], obviating the possibility for informed consent. Some persons may also resist the care that health professionals consider to be in their best interest due to symptoms of SMI, such as command hallucinations [63]. This could impact the voluntariness of their actions, so one can argue that they are not truly autonomous [64].

Therefore, while geographical variation in compulsory hospitalisation challenges the principle of respect for autonomy, from the point of view laid out in Principles of Biomedical Ethics, this applies only among patients who have decision-making capacity, understanding, and do not experience command hallucinations or other forms of more internally controlling conditions. For persons who are not considered autonomous within this framework, other principles, such as non-maleficence, can still be infringed and would be accorded more weight. We return to different perspectives on autonomy and approaches to facilitate autonomous decisions below.

### Beneficence

Compulsory admissions for treatment or prevention of harm to self are performed on the assumption that patients benefit from them. The principle of beneficence thus underlies the paternalistic justification for compulsory hospitalisation. Patients are expected to be better off because of the intervention, even if they do not want it, compared to a scenario without it. This is justified by conferring a benefit or avoiding or limiting harm.

The efficacy of compulsory hospitalisation has proven difficult to establish, not least due to ethical and legal concerns surrounding randomisation. Therefore, limited evidence is available to inform whether higher or lower levels of compulsory hospitalisation in an area is beneficial to patients with SMI or their peers.

A number of case control studies suggest that most patients improve somewhat as a result of compulsory hospitalisation, but there is also evidence that many patients show limited or no improvement [49, 65–68]. Evidence is lacking for the claim that compulsory hospitalisation is effective in preventing death by suicide [69, 70]. No reliable method for predicting who is likely to benefit, or not, from a compulsory hospitalisation has been established.

Systematic reviews of patient surveys demonstrate mixed experiences with compulsory hospitalisation. Relatively high shares of patients report positive views on their compulsory admission in retrospect and perceive it as beneficial [65]. However, a systematic review still found less treatment satisfaction among patients who had been hospitalised compulsorily compared to voluntarily [71].

Compulsory hospitalisations may be beneficial because they reduce the risk of harm to others. In such situations, there is less emphasis on expected benefit to the patient. Here other principles, particularly “the harm principle” [72], are likely to be given more weight. Some argue that such interventions, where the primary objective is not to benefit the patient, ought to be considered public safety measures rather than health care [73]. Others claim that interests beyond the patient’s own should be secondary when considering compulsory hospitalisation, but that it can be in the patient’s interest not to harm other people [74].

While the evidence for benefit from compulsory hospitalisation remains unclarified, it is difficult to decide what is best for patients. Hence, it remains complicated to identify whether, or to what extent, overuse or underuse infringes the principle of beneficence. This would also depend on the extent to which less restrictive courses of action have been attempted. If benefit resulting from a compulsory hospitalisation could also have resulted from voluntary initiatives, then any benefit should not be attributed to the coercive practice.

### Non-maleficence

The extensive literature documenting various ways that people experience harm related to involuntary hospitalisations raises concern [19]. Such harm has been categorised as emotional, cognitive, or physical [75]. Additionally, social and relational harm due to stigma, could affect patients as a result of hospitalisation. Therefore, overuse of compulsory hospitalisation would result in more such harm than necessary. Underuse could also result in unnecessary harm in cases where the patient does not receive required care or presents a risk of harm to themselves or others. In both cases, geographical variation in compulsory hospitalisation would represent different exposure to various kinds of harm, which means that the principle of non-maleficence would be infringed more in some areas than in others.

It is difficult to predict who might be harmed by compulsory hospitalisation and to what degree. Nonetheless, geographical variation suggests that populations are more exposed to the risk of harm in some areas than others, which infringes the principle of non-maleficence.

### Justice

The principle of justice implies that equal cases should be treated equally and unequal cases differentially. If the legal status of the hospitalisation depends on where the patient lives or is treated - for instance, due to differences in evaluating decision-making capacity [76], interpretation of legal criteria [77], or experience-based local practice [27] - then the resulting geographical variation would violate the principle of justice.

According to this principle, all citizens should have access to health services of the same type and quality regardless of where they live. Overuse of compulsory hospitalisation may indicate a lower quality of care [78], which would violate the ethical principle of justice. In principle, underuse of compulsory hospitalisation may also be unjust, particularly in the case of danger to self or others.

Ethical considerations surrounding health care delivery involve the equitable distribution of limited goods or benefits. Resources spent on one patient group can result in less resources available to another group. As discussed, it is unclear whether compulsory hospitalisation constitutes a good, an unfair disservice, or both, and opinions might also differ according to the perspective of patients, relatives, or society. Some stakeholder groups strongly disagree that compulsory hospitalisation represents beneficence. This complicates the analysis but can contribute to explain the diverging opinions on compulsory hospitalisation. In any case, unwarranted geographical variations in compulsory hospitalisation violate the principle of justice and are thus ethically challenging.

### A “right” level of compulsory hospitalisations?

From the analysis on how variation in the form of underuse or overuse of compulsory admission in different ways would violate the principles of respect for autonomy, beneficence, non-maleficence, and justice, the question of what represents the “right” or appropriate level of use appears to be central [79]. To determine what is too much or too little we need to define what is optimal. To disentangle the ethical dilemmas stemming from geographical variations and to improve care, we need to establish what provides the maximal benefit-risk ratio of involuntary admissions [80, 81].

The notion of “right care” is central to the study of geographical variation in health services [2]. The definition used in the Lancet series devoted to the issue is explicitly or implicitly based on the four ethical principles as discussed above: “What is right care? In its simplest definition it is care that weighs up benefits [beneficence] and harms [non-maleficence], is patient-centred (taking individual circumstances, values, and wishes into account) [autonomy], and is informed by evidence [beneficence], including cost-effectiveness [justice]” [82].

As asserted, there is disagreement surrounding the benefits of compulsory hospitalisations. Compulsory hospitalisations that are initiated in the best interest of the patient (for treatment or prevention of harm to self) appear in the literature to be more controversial than those aimed to prevent harm to others [49, 54, 83–85], and it has been argued that compulsory hospitalisation can only be justified when the patient lacks decision-making competence [54]. While some argue for the total abolishment of coercion [31], most stakeholders seem to agree that compulsory hospitalisation is likely to remain necessary in some cases to prevent serious harm [49, 86]. Health authorities in many jurisdictions strive towards reduced and appropriate use of coercion but, with lacking evidence and ongoing controversy, theoretical consideration concerning the possible existence of a “right” of compulsory hospitalisation is warranted.

What level is “right” is likely to vary between contexts but would never be higher than what is necessary. What is necessary would depend on several factors, some of which can be altered by policy makers. If there are few or no other available interventions, compulsory hospitalisation more easily appears necessary from the clinician’s perspective, than if a broader range of voluntary options existed [18, 21, 87, 88]. Therefore, compulsory hospitalisation that arises because of a lack of less restrictive interventions, in other words supply-driven [2] compulsory hospitalisation, should not be considered necessary, and would consequently not define “right care”, and not be ethically justifiable.

**Fig. 1:**
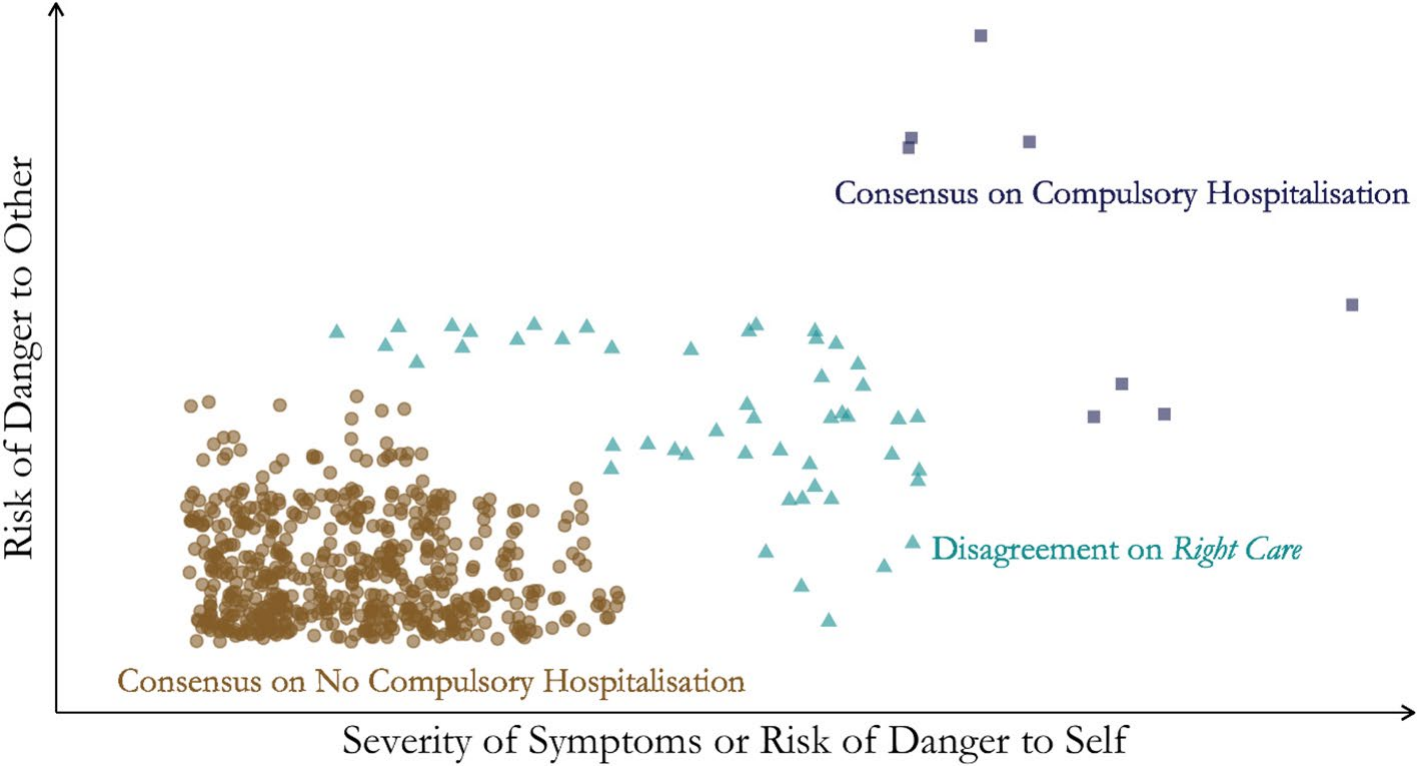
Disagreement on Right Care for Patients in Mental Health Care (but not for all patient groups). The figure is for illustration purposes and does not contain real data

If “right care” cannot be defined by empirical evidence, then professionals frequently apply experience-based consensus. Consider the conceptual model illustrated in Fig. 1, which is intended as a heuristic device. The dots represent patients with a mental health condition in acute need of hospitalisation, with severity of symptoms or risk of danger to self along the x-axis, and risk of danger to other along the y-axis. Health professionals tend to agree that most patients are not in need of compulsory hospitalisation, as illustrated by the brown circles. The grey squares represent a small share of patients that most health professionals would agree need compulsory hospitalisation, because of danger or treatment needs. The triangles represent the patients where there is clinical disagreement over best treatment. Disagreement can arise from uncertainty about decision-making capacity, diagnoses, danger, or physician-opinion on the use of compulsory hospitalisation. It is likely for this group of patients that the risk of being admitted involuntarily versus voluntarily differs considerably between areas, as observed in the geographical variation in rates of compulsory hospitalisation. This is because the patients in the other two categories are likely to be admitted according to the same legal status regardless of area. For patients in this group, moving from one of the areas with the highest level of compulsory hospitalisation, to one of the areas with the lowest level, might cause them to be received and treated differently. If this is due to factors such as the existence of superior, less restrictive forms of care in the low-coercion area, it will violate the ethical principle of justice.

In theory, there is a “right” level of compulsory admission, which can be described as those that are necessary, even if evidence lacks for which situations those are. Although it can be difficult to identify precisely in which situations compulsory hospitalisation is necessary, stakeholders tend to agree that such situations exist.^1^,^2^

If compulsory hospitalisations are used in situations where they are not necessary, it would constitute overuse. Such overuse would possibly violate all the four mentioned principles of health care ethics. It could also be unlawful [77]. If patients in the necessary situations are not compulsorily hospitalised, it would constitute underuse. This could violate their rights as patients, which would infringe on the principle of justice. Moreover, if their condition deteriorates because of a lack of health care, it would violate the principle of non-maleficence.

In situations where a crisis has already developed, it may be too late to attempt voluntariness, and professionals in acute mental health care may be required to handle such “unmanageable” situations through coercive interventions. Prior to crisis development, it is possible that sustained, high quality mental health services can reduce the need for compulsory hospitalisation in a geographical area through approaches that promote recovery or increased willingness to interact with services. Research from various countries shows that the use of compulsory hospitalisation can be reduced through targeted efforts and development of good practices that promote voluntariness [21]. The “right” level of compulsory hospitalisation would thus not be higher than those situations that are necessary over time within a health system with voluntary services of adequate quality. Consequently, it is important to develop health services that can encourage people to receive help voluntarily, even while in a severely affected state of mental health [90].

### Discussion with implications for service delivery

We have argued that unwarranted geographical variation in compulsory hospitalisation challenges basic principles of health care ethics, such as beneficence, non-maleficence, justice, and respect for autonomy. Our focus has been on geographical variations of compulsory admissions and not coercive practices in mental health care more broadly, where there are many ethical challenges that are discussed by others in detail [47–56].

When discussing the ethics of geographical variation, it seems morally relevant to consider the reasons why such variation manifests. In this final section we will therefore consider our analyses in the context of theory and empirical evidence, and discuss situations where the variation is related to the interests of the patient (treatment outcome, danger to self), the interests of others (danger to others), or professional or organisational differences.

Wennberg has shown that the medical procedures most prone to professional disagreement or controversy display the highest amount of geographical variation [2]. In contrast, conditions such as heart attacks or hip fractures display little geographical variation because physicians tend to agree on appropriate treatment for these conditions. Given the uncertainty surrounding outcomes of compulsory hospitalisation, situations can arise where it is neither clear if voluntary care is viable, nor evident if compulsory hospitalisation is indicated, as illustrated by the triangles in Fig. 1. In these situations, there is little agreement on what constitutes “right care”, and geographical variation might be expected. Theoretically, it seems reasonable that varying treatment philosophies or differences in clinical practice could result in higher or lower levels of compulsory hospitalisation in an area. For example, when facing a patient with newly developed psychotic symptoms and no history of SMI, one clinician might follow a strategy of early intervention and consider compulsory hospitalisation to be necessary at an early stage. On the other hand, a clinician who prefers “watchful waiting” and encourages voluntary follow-up outside of hospital may continue to treat according to interventions that are more tolerable to the patient. Contrasting treatment philosophies, experience-based local practices, or differences in clinical gaze among professionals facing a complex evidence base, could thus theoretically result in different levels of compulsory hospitalisation. This would infringe the principle of justice.

We argued that there exists a small share of situations in mental health care where there is a consensus among professionals that compulsory hospitalisation is the right intervention, as illustrated by the squares in Fig. 1. If this agreement is real, it would imply that compulsory hospitalisation is considered effective care [2] for the patients involved. A consensus is not a substitute for evidence of efficacy, however, of which the practice of insulin coma therapy is but one example [91]. It is therefore of paramount importance to clarify whether enforcing health care on patients is truly of benefit through continued research on compulsory hospitalisation, preferably including outcomes of relevance to patients, professionals, relatives, and society at large [56, 92].

Outcomes of compulsory hospitalisation are not identical for different patients or patient groups, and persons with certain conditions may be more likely than others to benefit, or become harmed, from admission. The current scarcity of evidence for beneficial outcomes of compulsory care is itself an ethical problem. The use of similarly invasive procedures is not likely to be considered in other fields of medicine on such a weak evidence base [93]. Importantly, if compulsory hospitalisations do not produce the benefits expected from the intervention, they cannot be considered necessary [94].

While the ethical principle of beneficence has been used as justification for compulsory hospitalisation on the assumption that it promotes the well-being of the patient, those who argue against compulsory hospitalisation tend to place more emphasis on the principles of non-maleficence and respect for autonomy. For persons who have been involuntarily hospitalised but experienced no benefit from the coercive practice, it can appear particularly troubling that what is perceived as benefits at a group level are used to justify what they perceive as harm to them.

It is also concerning that no “safety valve” exists for people who have had particularly harmful experiences of compulsory hospitalisation, if they are facing compulsory rehospitalisation. In other health services, patients who experience high maleficence can abstain from further care, regardless of the physician’s recommendation. Patients who are compulsorily hospitalised do not automatically have this option. Some stakeholder groups have therefore requested that a right to reserve oneself from coercive treatment be instated for patients who have previously been medicated involuntarily [95].

Geographical variations resulting from differences in evaluating decision-making capacity, interpretation of legal criteria, or uncertainty regarding diagnosis, were argued to violate the ethical principle of justice. Further work on best practices can inform guidelines and is likely to result in more reliable evaluations of whether compulsory hospitalisation is necessary. This will contribute to reducing overuse, underuse, and improve services.

### Different approaches to facilitate autonomous decisions

One of the most frequent complaints from individuals who have been involuntarily committed is the experience of not being listened to concerning decisions about their treatment [57]. This is perceived as a violation of integrity or dignity, which is connected to, and synonymous with, autonomy according to some theorists [96].

The CRPD states that “measures relating to the exercise of legal capacity [shall] respect the rights, will and preferences of the person… and [be] tailored to the person’s circumstances” ([23] p9). This implies that the wills and preferences of individuals with reduced decision-making capacity ought to be respected, including persons who do not fulfil all autonomy criteria of Beauchamp and Childress. This can, for instance, take the form of advance care planning, supported decision-making [97], or through preference for medication during detention [98, 99]. If patients are allowed to express their preference for a particular “treatment regime”, for example several shorter admissions to prevent deterioration, instead of one longer involuntary admission, resulting geographical variation would be more in accordance with the principles of respect for autonomy and non-maleficence.

Self-binding advance decision-making is one suggested approach [100, 101] which appears particularly relevant for individuals with “fluctuating” decision-making capacity [102]. While in remission, patients are encouraged to formulate wishes for their possible future manic or psychotic episodes, based on previous experiences with their illness and mental health services. By formulating such a Ulysses contract in stable periods when the patient has decision-making capacity, their autonomy is retained, even though their liberty will be restricted during periods of compulsory hospitalisation.

In this way, patient autonomy can be increased by extending the evaluation period prior to initiating a compulsory hospitalisation. It could also be increased by delaying invasive procedures and attempting other strategies first, such as Open Dialogue [103], which might help reduce psychotic symptoms and alleviate the perceived need to use coercion. Areas without such alternative interventions might infringe the principle of autonomy more than necessary.

Autonomy is better understood as a matter of degrees, rather than dichotomies, even within Beauchamp and Childress’ framework. Coercive interventions, such as compulsory hospitalisation, are sometimes temporarily justified with the aim of restoring or strengthening the autonomy of individuals who are considered to lack decision-making capacity [74]. As Beauchamp and Childress state: “…the criteria of the autonomous person and of the competent person are strikingly similar” ([58] p114). Some argue that restoring autonomy is the only situation where coercive mental health treatment is justified [104]. According to interview-based studies, coercive interventions are deemed more acceptable by patients if the practice is perceived as restoring their autonomy [105].

### Right level of compulsory hospitalisation

A mental health service system with no overuse or underuse, where compulsory hospitalisation is only used when truly necessary, and there would, thus, be no false positives or false negatives, could be claimed to have the “optimal level” of compulsory hospitalisation. Public reporting of geographical variation in compulsory hospitalisation can increase awareness of overuse and trigger reductive efforts in outlier regions. The systematic monitoring of coercive interventions with feedback to decision makers has previously been shown to reduce the use of seclusion and restraint [18].

While our ethical analysis and discussion provides no immediate solution to identify the “right” level of compulsory hospitalisation, it shows that compulsory hospitalisations which arise from a lack of less restrictive initiatives are not ethically justifiable. One possible strategy for estimating the “right” level of compulsory hospitalisations could be to base it on observed population health outcomes. Following the reasoning of O’Reilly et al. [106] we can hypothesise that a lower threshold exists for rates of compulsory hospitalisation, below which negative effects will appear in a geographical area. Relevant outcomes used to identify this minimum rate could be suicide rates; all-cause mortality rates; rates of violent crime committed by persons with SMI; rates of incarceration of persons with SMI, as well as the level of burden on care givers. This threshold would be the minimum level of compulsory hospitalisation required in an area which could be considered responsible. Use below this rate would be underuse and use above this threshold would be considered unnecessary so would constitute overuse. Therefore, it can be argued that the “right” level would be the minimal necessary care to avoid negative outcomes for the patients and the wider community.

### Lack of less restrictive interventions

Services that are contingent on local capacity, and not on the patient’s needs or preferences, are labelled supply-driven care within Wennberg’s framework of types of health services [2]. If organisation of mental health services at a local level can be shown to promote voluntariness and prevent the need for compulsory hospitalisation, an uneven distribution of less restrictive initiatives is likely to constitute an ethical challenge. As we have shown previously, there were lower levels of compulsory hospitalisation in areas that had more general practitioners per capita in Norway 2015–2018, and an increase in general practitioners per capita was associated with significantly lower levels of compulsory hospitalisation within those areas [107]. This suggests that compulsory hospitalisation is partly supply-driven. Variations in supply-driven services are ethically problematic, as they do not relate to patients’ preferences or needs but are the result of the dimensioning and organisation of services. Service user researchers often focus on unmet needs - a “type of support that is desired but may not be available” ([18] p23) - which may help avert compulsory hospitalisation. Professionals in primary mental health services in Norway suggested that improving everyday life for persons with SMI or addiction challenges, such as living conditions and employment support, was conducive to reducing the need for compulsory hospitalisation [108]. Continued research into community health services that could prevent the need for compulsory hospitalisation to develop in the first place is therefore crucial.

Identifying effective and good practices that reduce the need for compulsory hospitalisation will improve mental health services [109] and likely reduce geographical variation. One example of services only offered in certain areas is Assertive Community Treatment, which has been documented to reduce frequency [20] and length [110] of compulsory hospitalisation. Preventing the need for compulsory hospitalisation to develop in the first place would allow patients to maintain their autonomy whilst simultaneously averting possible harm from coercion.

Compulsory hospitalisation is not only controversial, but it also creates the possibility for the use of other coercive practices with uncertain outcomes, such as forced medication, mechanical restraints, seclusion, or outpatient commitment, that would be less likely to have been initiated without the admission. There is therefore reason to believe that geographical variation in compulsory hospitalisation concurs with geographical variation in other coercive practices [27]. This can have serious consequences for individual patients if they are treated unfairly. The use of forced medication appears to be at least as controversial as compulsory hospitalisation and it has been argued that medication-free alternatives ought to be instated - especially considering that a proportion of patients do not seem to benefit from anti-psychotic medication [111]. It is likely that the existence of such alternatives could increase the willingness to engage voluntarily with mental health services, particularly for patients with previously negative experiences. Experiences from Heidenheim in Germany suggest that a service system practically free of coercion is possible in practice [90].

Professionals are generally unaware of overuse of health services due to supply-driven health care [2]. In highly specialised and disjointed services, physicians in an emergency department might rarely be provided with feedback on whether they refer patients to compulsory hospitalisation more often than other colleagues. A study of a Norwegian emergency unit that had considerable success in reducing the number of compulsory referrals suggested that personal feedback was the strongest driver of the reduction [112]. Considering this alongside other reports of successful efforts at reducing coercive practice [113] might indicate that overuse is not uncommon.

Clinicians report that patients are sometimes referred to compulsory hospitalisation on the assumption that there are few available hospital beds, and that a compulsory referral is more likely than a voluntary referral to result in hospitalisation [114]. Such cases would constitute a supply-driven use of coercion and would not be ethically justifiable. This is concerning in light of the continued reduction of beds in mental health institutions observed internationally for decades [115, 116].

### Admission criteria

Since we cannot precisely identify the situations in which compulsion is necessary and thus the “right” level, one can argue that it is better to aim below, rather than above, since overuse appears to be more ethically problematic than underuse. Indeed, the current policy drive towards reduced use of compulsion [117, 118] suggests that overuse of compulsory hospitalisation is considered a more prevalent problem than underuse. This can be related to the ethical principles, since overuse involves the potential violation of all four principles, while this is not necessarily the case for underuse.

However, when considering danger to others, underuse can result in considerable harm. This has been seen to cause newspaper discussions to call for increased use of compulsory hospitalisation following serious crimes committed by persons with SMI. This conundrum highlights the tension and conflicting interests under current legislations - human rights discussions pull towards reduced levels of compulsory hospitalisation for treatment, while protection of society from persons with SMI and a high risk of violence continues to be part of mental health services’ policing function. The “right” level of compulsory hospitalisation may well differ with admission criteria. In countries with multiple criteria for compulsory hospitalisation, it could be worthwhile to separately investigate the geographical variation among persons committed out of concern for others and admissions for treatment or prevention of harm to self [49].

Risk assessment is challenging due to the low rate of events and high likelihood of false positives [119]. As highlighted by the CRPD, there could also be an inherent injustice in the way danger due to mental illness is handled versus other dangerous situations that do not involve mental illness [120]. This could also perpetuate stigma towards persons with mental health disabilities.

Finally, when contemplating the “right” level of compulsory hospitalisation it is relevant to compare rates between jurisdictions and consider the fact that some countries appear to manage with considerably lower rates of compulsory hospitalisation per population [25]. Similarly, it is also necessary to consider the existence of exceptional catchment areas with very low or non-existent rates of compulsory hospitalisation [90]. While low rates can be due to low prevalence of SMI, it is also possible that they result from health services with a high rate of recovery. If it can be demonstrated that similarly low admission numbers are possible elsewhere this will strongly alter the numbers of the so-called “right level”.

### Limitations

While we restricted the analysis of principles to the four traditional principles of health care ethics, other principles could also be relevant in this context. These include: respect for privacy; solidarity; respect for integrity; sanctity of life; efficiency [121], and vulnerability [122].

The focus of this study is geographical variation in compulsory hospitalisation, but many of the challenges identified and discussed are relevant to variation in other types of compulsory interventions – both diagnostic and therapeutic. For example, the use of mechanical restraints, seclusion, forced medication and outpatient commitment. However, these warrant special attention since the ethical considerations would not apply equally to all these cases.

## Conclusion

Compulsory hospitalisation represents an unusual type of health service since it is not based on consent, restricts patients’ liberty, and is experienced as harmful by many. Therefore, geographical variation in compulsory hospitalisation poses special ethical challenges. The ethical principles of respect for autonomy, non-maleficence, beneficence, and justice, are all potentially infringed by unwarranted geographical variation in compulsory hospitalisation. Uncertainty regarding outcomes of compulsory hospitalisation is likely to preserve geographical variation and further research on outcomes for different patient groups and admission criteria is needed. Geographical variation in compulsory hospitalisation can be contingent on local capacity (supply-driven) but can also be the result of physician-opinion; both sources of variation can be considered ethically challenging. The “right” level of compulsory hospitalisation might differ with admission criteria but would never exceed the level that is minimally necessary over time within a health service system that promotes voluntariness through good practices. Standardised guidelines for assessing decision-making capacity, diagnosing, and interpretation of legal criteria ought to be established or continued to reduce geographical variation and increase adherence to the principle of justice.

## Data Availability

All data generated or analysed during this study are included in this published article.

## Footnotes

1 This might explain why legislative changes designed to reduce compulsory hospitalisation seem to have limited impact on observed rates [49, 89].

2 Although most stakeholders agree that compulsory hospitalisation can be necessary in certain situations, there is ample disagreement concerning the content of such hospitalisations, like the use of forced medication.
